# 3D Printing Techniques for Dental Prosthetic Models: A Narrative Review and Contemporary Perspectives

**DOI:** 10.7759/cureus.103077

**Published:** 2026-02-06

**Authors:** Panagiotis Galiatsatos, Aristidis Galiatsatos

**Affiliations:** 1 Biomedical Sciences - Division of Dental Technology, University of West Attica, Aigaleo, GRC

**Keywords:** 3d printing, additive manufacturing techniques, cad-cam dental systems, digital dental models, narrative review

## Abstract

Over time, the methods for creating dental models have expanded significantly. Nowadays, digital technology and computers in medicine offer more options beyond plaster casts made using the traditional method. The advancement of computer-aided design and computer-aided manufacturing (CAD/CAM) systems provides a broad array of technologies, including both subtractive and additive techniques. The main purpose of this paper is to present the various modern three-dimensional printing systems concerning the fabrication of dental models and subsequently compare them both with each other and with the traditional technique of plaster model fabrication. A description of the main methods of additive manufacturing (AM; also referred to as 3D printing) for dental models is provided, along with bibliographic data regarding their accuracy and effectiveness, as well as comparisons with traditional manufacturing methods. The results of this paper indicate that stereolithography (SLA), digital light processing (DLP), and PolyJet technologies are the most precise choices for producing full arch dental models for prosthodontic use, offering high levels of trueness. Using digital tools and software, AM creates desired casts layer by layer, streamlining the production of complex, customized dental models with high speed, accuracy, and lower costs. The clinical significance of 3D-printed dental models is multifaceted, offering improvements in accuracy, efficiency, communication, education, cost-effectiveness, and innovation in dental practice. These technological advancements contribute to providing higher-quality care and better outcomes for patients.

## Introduction and background

In today's era, as in many other industries, there has been a rapid trend towards digitalization in dentistry, with the use of computer-aided design and computer-aided manufacturing (CAD/CAM) technologies becoming increasingly common. Hence, the application of digital technology in dentistry is continuously increasing. With the current advancements, the future of dentistry is unquestionably digital. Advanced digital solutions for impressions, treatment planning, restoration design, and additive manufacturing (AM; also referred to as 3D printing), which were once prohibitively expensive, are now becoming more accessible, transforming numerous dental practices worldwide. As CAD/CAM systems gradually replace traditional methods and become central to dental care, digital solutions are becoming indispensable for every business in the dental industry.

Three-dimensional manufacturing techniques are classified into additive methods (AM) and subtractive methods (SM) [1-4]. In SM, material is cut from a solid industrially prefabricated block, with the final goal being the remaining part constituting the final construction. In AM, an object is produced by the sequential addition of material in hundreds or thousands of layers in a two-dimensional (2D) plane until its completion. There are various classifications of the techniques used in the three-dimensional production of objects. The American Section of the International Association for Testing Materials (ASTM) International Standard Organization creates voluntary consensus standards for a broad spectrum of materials, products, systems, and services. The ASTM Committee F42 on Additive Manufacturing (AM) Technologies has identified seven categories of AM: stereolithography (SLA), material jetting (MJ), material extrusion (ME) or fused deposition modeling (FDM), binder jetting, powder bed fusion (PBF), sheet lamination, and direct energy deposition [5,6].

The inventor of AM (also referred to as 3D printing) was Charles Hull of the University of Colorado in 1986 [7]. This researcher used ultraviolet light to harden the surface of overlays and named this process SLA [7,8]. That same year, Carl Deckard from the University of Texas introduced the selective laser sintering (SLS) technique [9,10]. In 1989, Scott Crump invented the technique of FDM [11]. Since then, a plethora of new techniques and variations have been introduced, corresponding to the evolution of printing devices and the available materials for use with these techniques.

However, the main techniques for printing materials to create models in prosthetics and dentistry, in general, are as follows: SLA, digital light processing (DLP), liquid crystal display (LCD) printing, and FDM, which includes two subcategories: fused filament fabrication (FFF) and PolyJet photopolymer (PPP) technology [6,12-15].

The rapid adoption of intraoral scanners in dentistry over recent years has fundamentally transformed clinical and laboratory workflows, enabling faster and more accurate digital capture of dental structures. However, this increasing reliance on digital data underscores the need for fully validated and reproducible dental model production pathways to ensure accuracy, reliability, and clinical applicability.

The primary aim of this paper is to explore the different contemporary AM (also referred to as 3D printing) systems used for fabricating dental models and to compare these systems with one another, as well as with the traditional plaster model fabrication technique. Τhis review focuses on clinical applications of AM for prosthodontic and orthodontic dental model production. English-language studies published between 2015 and 2025 were included to capture contemporary developments in printer technologies, materials, and digital workflows.

## Review

Methodology

This narrative review was conducted through a non-systematic search of the English-language literature published between 2015 and 2025. Databases including PubMed, Scopus, and Web of Science were searched using combinations of keywords, such as “additive manufacturing techniques”, “digital dental models”, “3D printing”, “dentistry”, and “CAD-CAM dental systems”. Original research articles, systematic reviews, meta-analyses, and consensus statements relevant to clinical dental applications were included. Emphasis was placed on studies reporting diagnostic performance, clinical validation, and applicability to routine practice. Seminal earlier publications of high relevance were also included to provide historical and technological context. Studies focusing exclusively on industrial applications, purely mechanical testing without a clinical context, conference abstracts, editorials, and non-peer-reviewed articles were excluded. Overall, 45 relevant publications were identified and included in the present narrative review.

Three-dimensional printing systems

Stereolithography (SLA)

**Figure 1:**
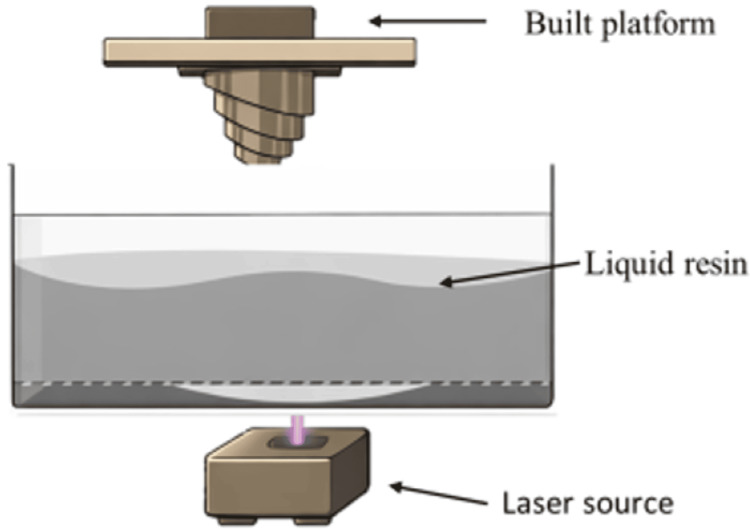
Schematic illustration of how an SLA printer works. SLA: stereolithography

In the SLA technique, the system includes a build platform, a vat of liquid resin, and an ultraviolet (UV) laser that cures and polymerizes the resin (Figure 1).

**Figure 2:**
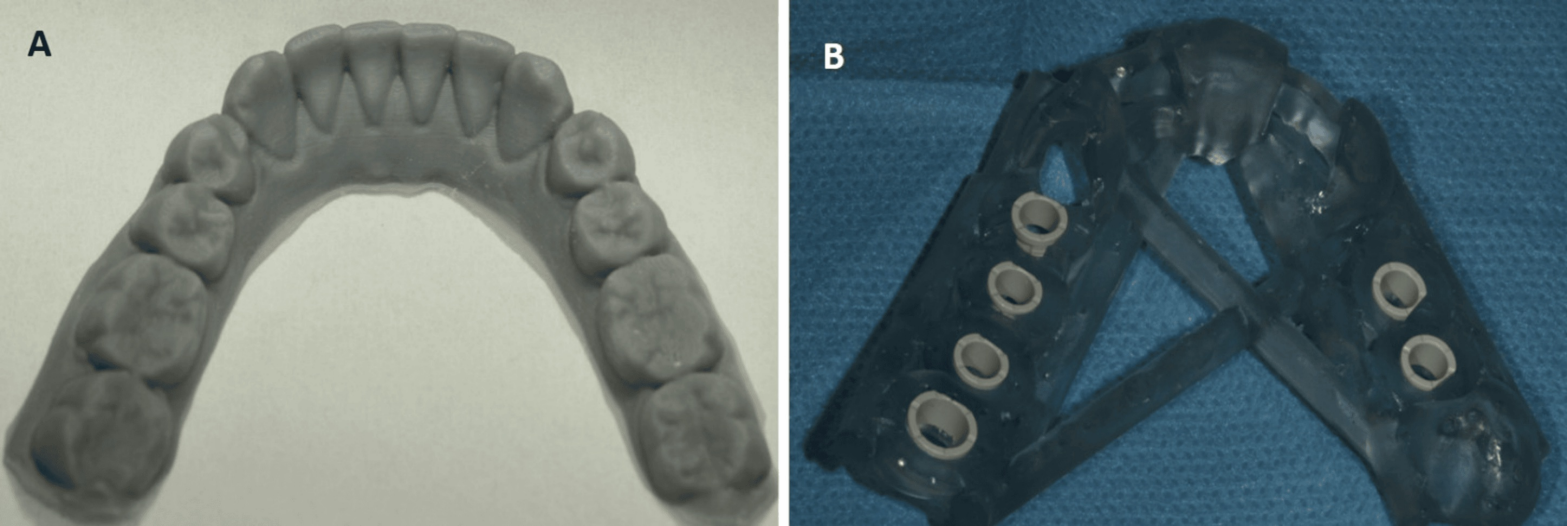
Stereolithography (SLA) 3D-printed constructions. (A) Cast and (B) surgical implant guide

This technique relies on a photopolymerizable resin in liquid form, which is polymerized layer by layer using UV radiation from a laser source [2,3,12,16,17]. The polymerization induced by the UV light occurs only on the surface of the material, so the layers are polymerized and bonded successively to form the three-dimensional object. After the construction process is finished, the object is submerged in a solvent bath to eliminate any excess or non-polymerized resin, and then it is placed in a UV oven to finalize the polymerization. Depending on the complexity and size of the object, SLA can take anywhere from a few hours to over 24 hours to produce the final product. The thickness of each layer and the wavelength of the emitted radiation depend on the device used. Moreover, the speed and duration of the emission are crucial in determining the depth of polymerization of the material and the thickness of each layer. The typical characteristic resolution ranges from 50 to 100 μm, and the wavelength of the UV radiation ranges from 200 to 500 nm, with an average layer thickness of 50-200 μm [17-20]. This technique has numerous applications in various fields, such as designing cranial, maxillofacial, and neurosurgical procedures; creating replicas of human anatomy; manufacturing surgical guides and temporary crowns and bridges; creating resin models for casting; orthodontic devices (including clear active and retentive aligners); complete dentures; dental models; custom trays; aesthetic diagnostic templates, and more (Figures 2A, 2B).

Digital Light Processing (DLP)

**Figure 3:**
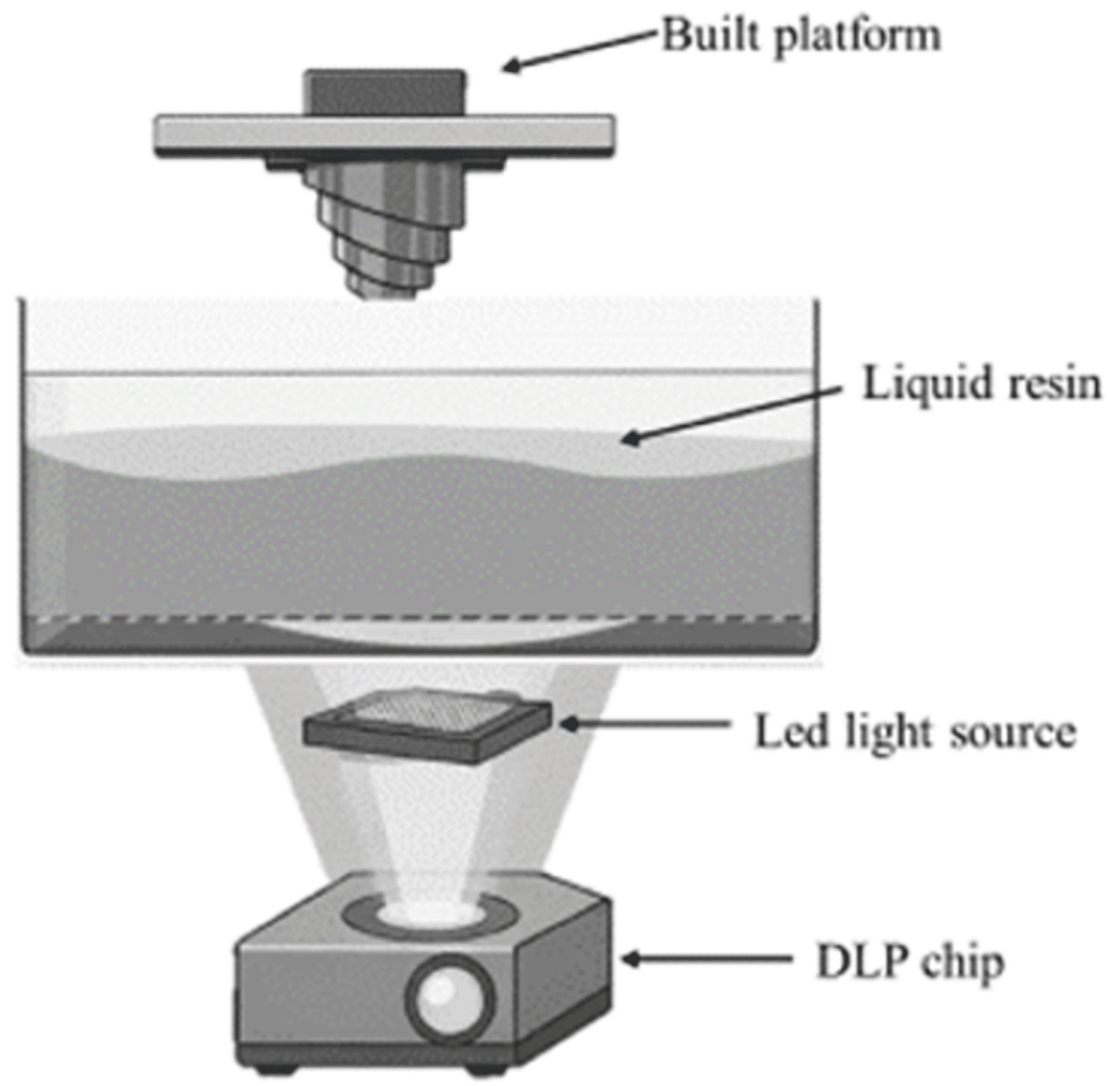
Schematic illustration of how a DLP printer works. DLP: digital light processing

DLP is a technology that utilizes a digital device equipped with micromirrors to project light. Its energy source is a conventional UV light source, which polymerizes the liquid resin into solid three-dimensional objects [6,12,17,21-23]. In essence, it employs a projector, similar to those used for office presentations, to cast an image of each layer of the desired object onto the liquid resin. A high-power LED light emitting in the UV range is specifically used to polymerize liquid photopolymer resin layer by layer. This process is managed by hundreds or even thousands of moving micromirrors, each directing the reflection of the incoming light. Each pixel of the image is associated with a single micromirror, which adjusts its orientation by a few degrees relative to the light beam's axis. Due to the capability of simultaneous polymerization of all points of a given cross-section, the DLP technique significantly reduces the material construction time (Figure 3).

**Figure 4:**
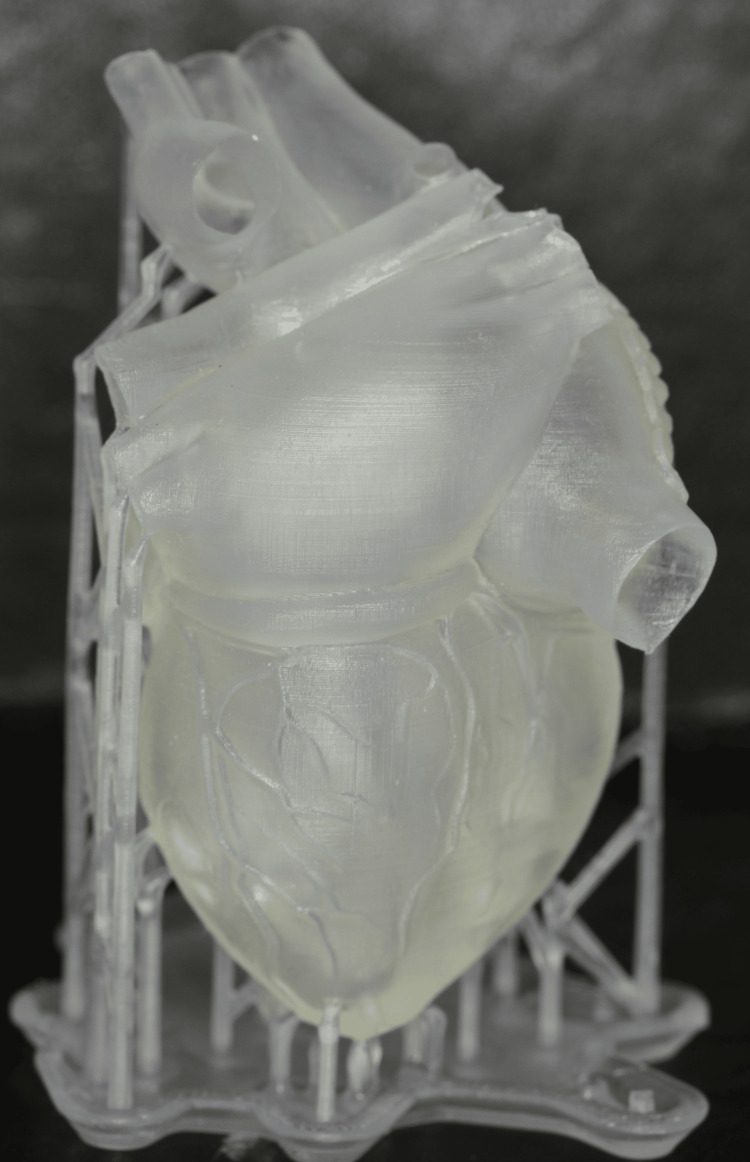
Human heart 3D-printed using DLP technology. DLP: digital light processing

The DLP technique is used in medicine and tissue engineering to create samples with predefined internal structures for complex organs, such as the trachea, heart, lungs, and blood vessels, achieving satisfactory biomechanical properties (Figure 4).

This demonstrates the high precision of its products. Additionally, it is used in dentistry for various applications, such as models, cast structures, temporary restorations, bases for removable dentures, custom trays, production of acrylic teeth for complete and partial dentures, and the creation of silicone matrices for temporary testing and permanent production of resin veneers [6,12,22].

LCD Printing Technique

**Figure 5:**
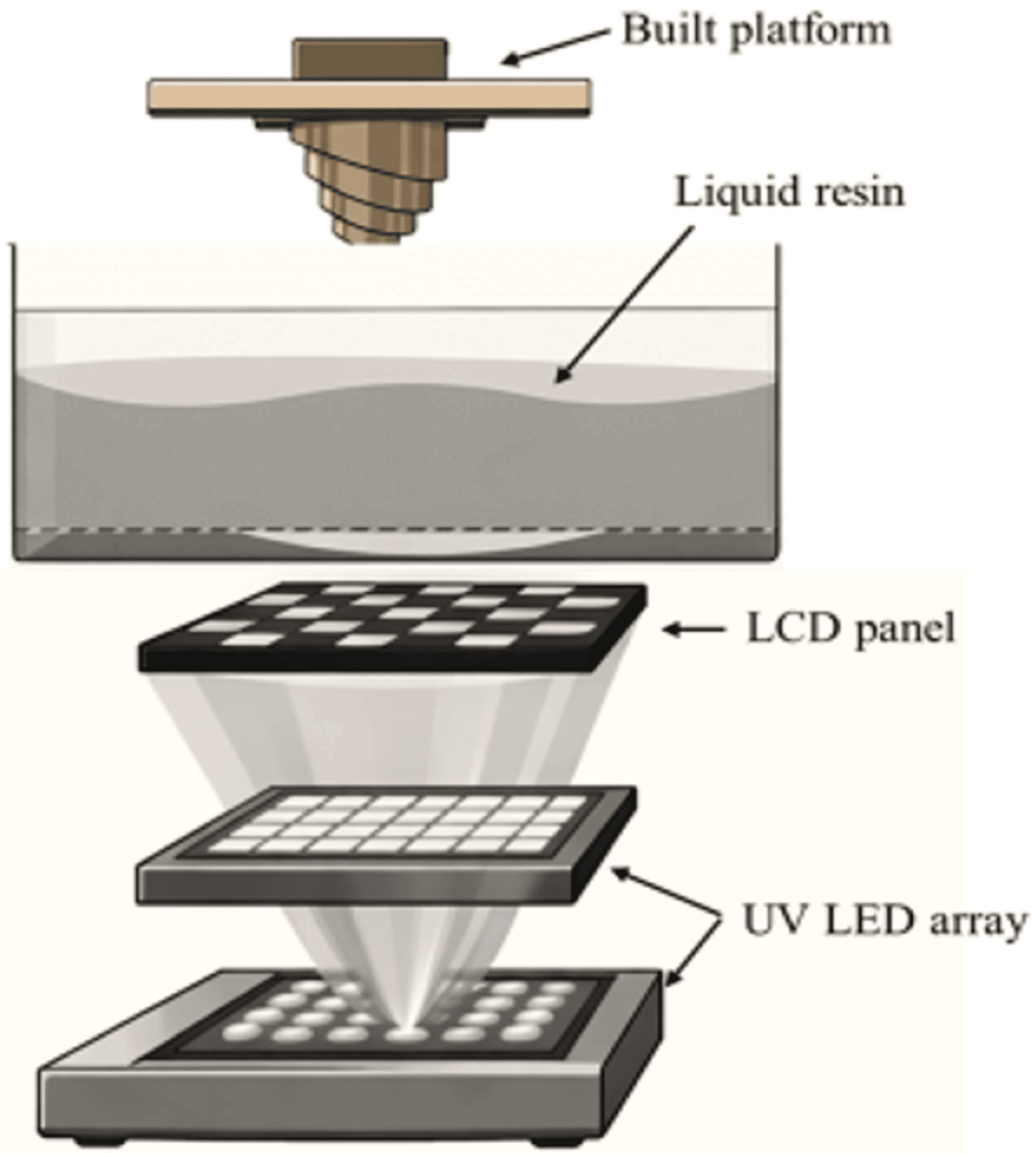
Schematic illustration of how an LCD printer works. LCD: liquid crystal display

In all three-dimensional printing technologies that use photopolymerization - ranging from SLA (laser scanning) to DLP and the latest LCD printing technology - the primary difference is found in the light source and imaging system, whereas the control system and printing process exhibit only minor variations. The key difference between DLP and LCD AM technology is found in the imaging system. Printing with LCD is based on a light source, typically LED type, emitting UV radiation [23-26]. The LCD screen is used to shape this radiation into specific patterns per layer, according to the desired geometry of the object being printed. The screen acts as a "mask," regulating the projection of UV light and exposing only the pixels required for the current printing layer. Therefore, unlike SLA and DLP, there is no need for a special device to direct the light (Figure 5).

Additionally, unlike the SLA technique where the solidification of the photopolymer takes place under exposure to a focused UV light source, LCD printers use a low-power LED light source (wavelength: 400 nm), which passes through the thin LCD panel that functions as an optical shutter for the light source, exposing the entire underlying layer.

**Figure 6:**
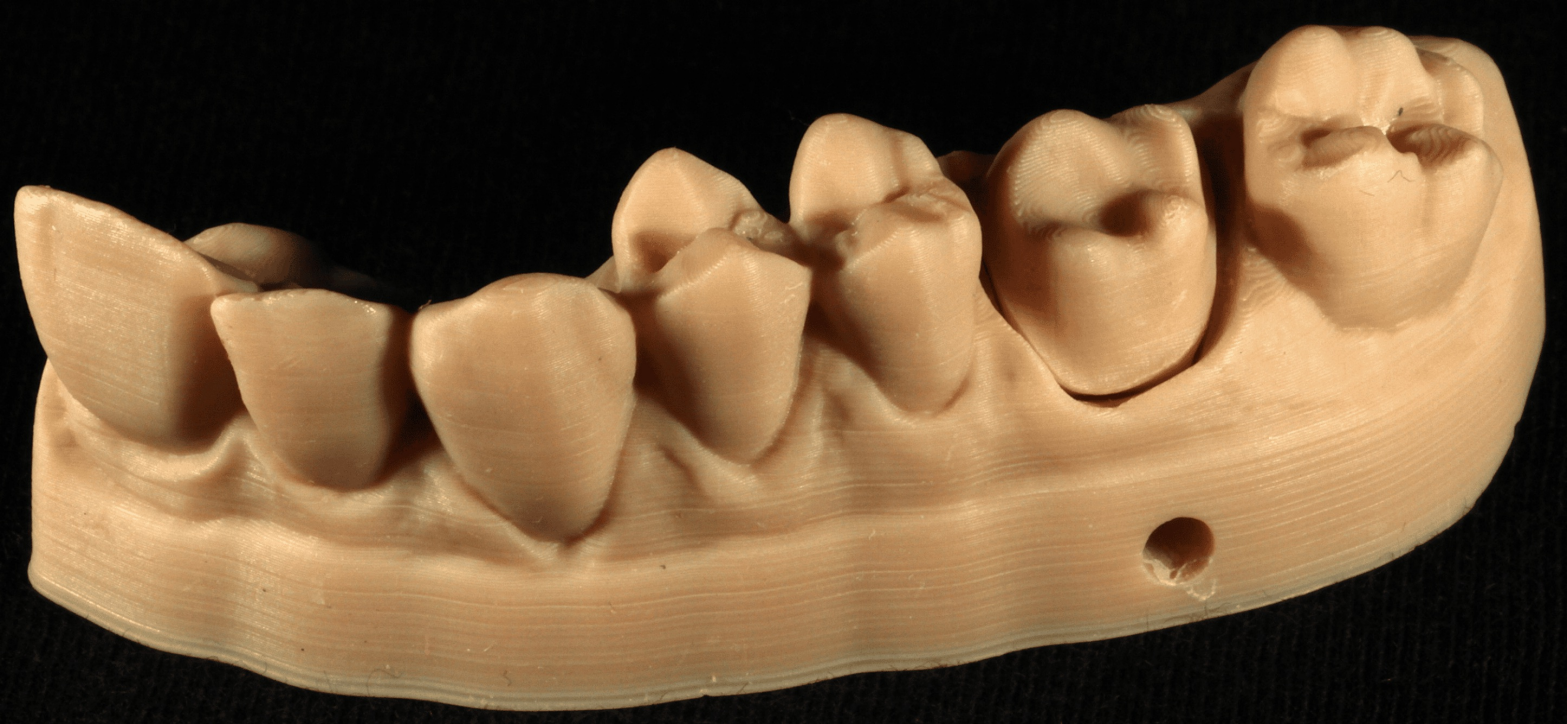
Cast printed with LCD technology. LCD: liquid crystal display

LCD printers are particularly cost-effective because they utilize less expensive components compared to 3D DLP printers, making them a more affordable option for resin AM. This has led to their application primarily as desktop printers, with numerous applications in both the medical/dental fields and the industry in general [6,12,23-26] (Figure 6).

Fused Deposition Modeling (FDM)

FDM technology was developed by Scott Crump in the late 1980s and subsequently popularized by Stratasys (a company headquartered in Eden Prairie, MN). The company acquired the trademark for FDM in 1990 [27,28]. The first production of a medical model using this technology was in 1999. Today, it is the most widely used printing technology in both dentistry and medicine. Finally, it is among the fastest-growing AM methods due to its lower cost, increased user-friendly operation (filaments versus powder), and reduced risk of contamination or material degradation [28-32].

FDM is a rapid prototyping method where a thermoplastic material is extruded layer by layer from a heated nozzle, operating under precisely controlled temperature conditions. This process resembles the operation of a "robotic glue gun." This technique is based on heating (up to approximately the melting point) a thermoplastic polymer filament or a metal wire, which is fed from a spool through an extrusion nozzle, and deposited onto a build platform. The entire system functions within a chamber maintained at a temperature slightly below the material's melting point. Precise control and maintenance of temperature during material deposition are crucial for the method's success. The straightforward nature of the technique enables the use of a wide variety of materials, including thermoplastics (waxes and/or polymers in filament form), resins, ceramics, and zirconia.

The FDM technique has two subcategories/variations: FFF and PPP technology [27-32].

The FFF technique is an AM process where a thermoplastic material is pushed through a heated nozzle to build up an object. Once the first layer is deposited, the build platform lowers by the thickness of one layer, making room for the addition of the next layer. This layering process repeats until the object is fully formed. The most frequently used thermoplastics in FFF printing are polylactic acid (PLA) and acrylonitrile butadiene styrene (ABS). PLA is recognized for its superior detail resolution, whereas ABS is valued for its durability.

PPP technology is an AM method that operates in a manner similar to an inkjet printer. It creates parts by depositing thousands of photopolymer droplets onto a build platform, which are then cured and solidified using UV light. The process begins by heating the photopolymer resin in a container until it reaches the appropriate viscosity. Once prepared, the printing process begins with the carriage moving along the X-axis of the build platform. The print heads selectively deposit the resin onto the platform, which is then cured by UV light. After each layer is completed, the build platform lowers by the thickness of one layer, and this process repeats until the entire object is fully printed.

**Figure 7:**
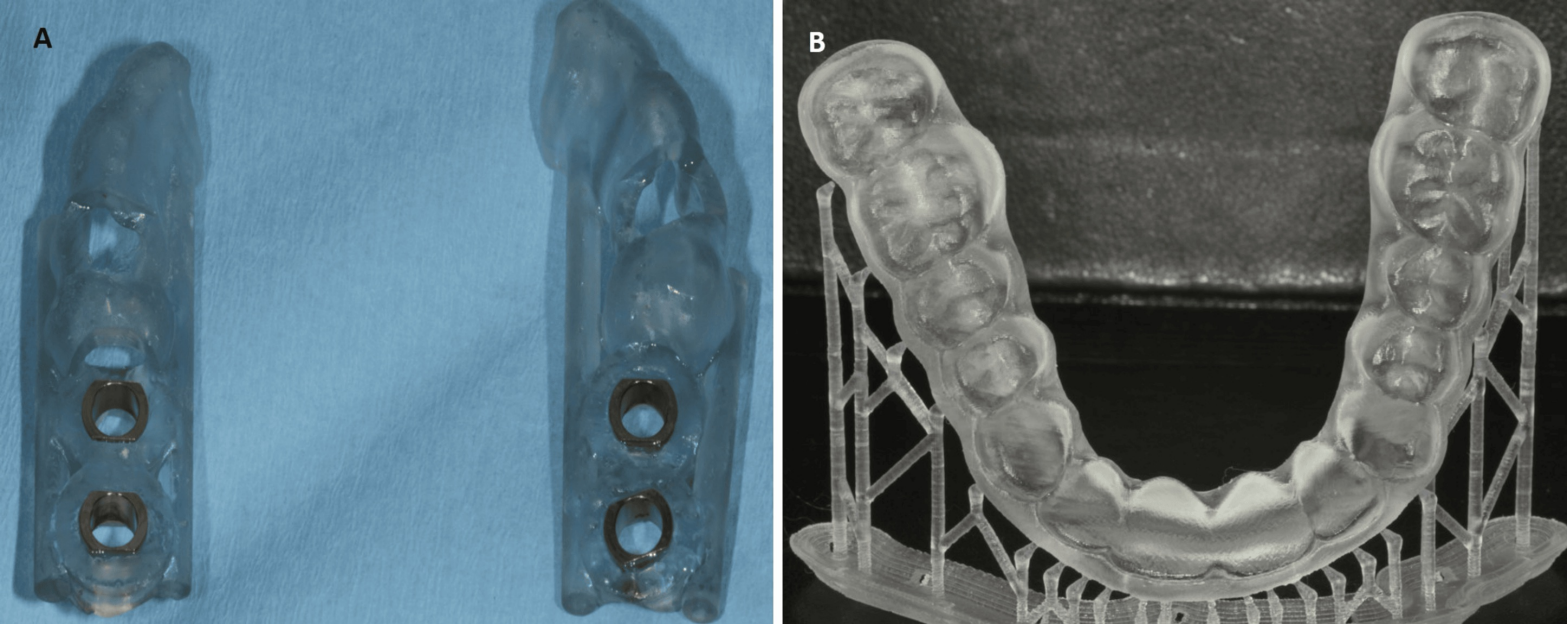
Fused deposition modeling (FDM) 3D-printed constructions. (A) Surgical implant guide and (B) whitening tray

Generally, the applications of the FDM technique are numerous (Figures 7A, 7B).

It can be utilized to fabricate prototypes of metal frameworks for partial dentures and prototypes of frameworks for fixed prosthetic works. Using the same technique, models and removable dies are printed, upon which resin patterns will later be created for casting in the fabrication of cast prosthetic works. Beyond dentistry, FDM devices are primarily used in the printing of prototype pilot constructions before their final application by industry and in medicine for creating original porous structures in tissue engineering and organ bioprinting [6,12,18,22].

Production process of casts

The production process of casts using AM begins with digitally scanning the patient's mouth and creating CAD files. These files are then used for the design and preparation for printing. The printing is carried out using appropriate devices and materials, and after the completion of the printing, the casts typically undergo post-processing to ensure their accuracy and construction quality. All types of casts used in dentistry can be produced with the AM method. Such casts include casts for diagnostic purposes, casts for fixed or removable prostheses, definitive casts for implant-supported prostheses, and orthodontic casts [6,12,15,16,21].

AM materials are typically classified based on the printing technologies they are associated with. The most common technologies include vat polymerization methods, such as SLA, DLP, and LCD, which employ liquid photopolymers, such as acrylates and epoxides. The FFF technology employs materials such as PLA and ABS. PPP technology utilizes liquid photopolymer resins, primarily acrylates [22,26,33].

Discussion

The adoption of digital technology in dentistry is rapidly expanding, with a focus on techniques based on three-dimensional design models, which have applications in modern dental prosthetics. Having a precise and reliable cast that accurately captures the dental arches, teeth, and surrounding tissues is crucial for the treatment plan of any prosthetic case. Therefore, in today's "digital" era, the conventional method of producing plaster casts is being challenged in favor of finding the most cost-effective, fastest, and optimal method of cast production through rapid printing technologies. Currently, a multitude of AM technologies are flooding the market, providing alternative options for the production of dental casts. The main technologies among these are SLA, DLP, LCD, FFF, and PPP. The aim of this review is to assess contemporary AM techniques for producing casts used in both fixed and removable prosthetic work. It will be a valuable resource for dental professionals, researchers, and academics who are interested in the integration of advanced manufacturing technologies into dental practice.

A key and primary factor in evaluating all these systems is the accuracy of the final product. However, many different factors influence accuracy. These include the materials used, the manufacturer, setup parameters, software, light source speed, light source intensity, emission angle, model build direction, number of layers, exposure time of each layer to the energy, amount of support material, and post-processing [6,34,35].

In 2014, Hazeveld et al. [36] performed a study comparing the precision of DLP and PPP printers. They discovered that the PPP printer exhibited greater accuracy compared to the DLP printer. Although the dental casts produced by the tested CAM systems may be reliable for orthodontic purposes, they might not meet the accuracy standards required for prosthodontic applications. In another study, Brown et al. [37] compared the accuracy of DLP and PPP models against each other, as well as with the conventional method. The findings revealed that all types of models showed high reproducibility across all measurements. Furthermore, there was a high level of consistency across all trials and measurements, except for crown height measurements when comparing plaster models to DLP models, where the average difference was statistically significant.

Comparative studies directly evaluating FDM/FFF versus photopolymer printers provide practical accuracy data for clinical decisions. Jaber et al. [38] evaluated the dimensional accuracy of dental models produced using two different 3D printing techniques - FDM and DLP - for potential use in the fabrication of clear orthodontic aligners. Twenty pairs of pretreatment plaster orthodontic models were scanned and reproduced using both printing methods. Forty linear measurements based on predefined reference points were taken from the original plaster models and their 3D-printed replicas. The results indicate that both FDM and DLP technologies can produce dental models with sufficient accuracy for diagnostic and analytical purposes. However, due to minor dimensional discrepancies, the use of these printed models for fabricating clear aligners may not fully guarantee optimal intraoral fit. Consequently, the authors recommend caution when using 3D-printed models for aligner fabrication and suggest the use of preliminary "zero aligners" to compensate for potential inaccuracies.

In another study, Maroua et al. [39] investigated the accuracy and reproducibility of linear measurements obtained from cone-beam computed tomography (CBCT)-derived digital dental models compared with conventional plaster models. Twenty-five patients who had CBCT scans taken for diagnostic purposes were included. Plaster models were fabricated from alginate impressions, while digital models were generated by segmenting the dental arches from CBCT data. Fourteen linear measurements related to arch length and width were performed on both model types. The findings indicate that CBCT-derived digital models provide a high level of accuracy and reproducibility for linear measurements. Despite minor discrepancies, these models appear suitable for clinical orthodontic diagnosis and treatment planning, offering a viable alternative to conventional plaster models.

Kim et al. [40] conducted a study comparing laser-SLA, DLP, FFF, and PPP printers, finding that the PPP printer demonstrated the highest precision, followed by the DLP, SLA, and FFF printers. Regarding trueness, the PPP printer also ranked highest, followed by the SLA, DLP, and FFF printers. In another study, Park et al. [41] found that casts made using the conventional method exhibited the least volumetric shrinkage compared to 3D-printed casts. Notable differences (p<0.05) were identified among the different types of 3D printers (DLP-UV, DLP-UV-LED, Polyjet), with the DLP printer using UV light showing the smallest shrinkage. Additionally, colored 3D visualizations revealed similar distortion patterns across all 3D printers. In a more recent study, Giudice et al. [42] evaluated the precision of LCD printers with laser SLA printers and concluded that, although LCD printers are less accurate, the difference is not clinically significant and does not affect the final outcome. Revilla-León et al. [43] examined the positional accuracy of implant analogs on 3D-printed polymer casts versus traditional dental stone casts, using a coordinate measuring machine (CMM) for evaluation. Their findings indicated that AM technologies could replicate a conventional definitive implant cast with accuracy comparable to traditional methods. 

In 2022, Tsolakis et al. [12] performed a systematic review to evaluate the literature on the accuracy of different types of 3D printers and the factors that may affect the AM (also referred to as 3D printing) of dental models in orthodontics. Their findings revealed that the accuracy of a printed dental cast can be influenced by the type of 3D technology employed, the design of the dental cast's base, and the materials used for printing. The review concluded that all types of 3D technologies are capable of producing clinically acceptable results for orthodontic applications.

In another recent study, researchers carried out a systematic review combined with a network meta-analysis to assess the accuracy of 3D-printed dental casts in comparison to digital reference models, utilizing data from multiple studies [44]. The results of this meta-analysis endorse the use of AM technologies for creating 3D-printed dental casts. For applications related to prosthetic and implant restorations, only SLA, DLP, and PPP technologies were identified as clinically acceptable for the precise production of full-arch casts. From the standpoint of orthodontics, FDM/FFF technologies were considered suitable. Additionally, a layer thickness of 0-50 μm was found to be ideal for achieving clinically acceptable precision for both prosthodontic and orthodontic uses. Besides accuracy, another important parameter in AM is the printing speed and, consequently, the device's performance [12,40-45]. The printing speeds of SLA, LFS, and DLP printers are generally comparable to each other. DLP 3D printers have a uniform printing speed because the projector exposes each layer of the entire object simultaneously, making the speed dependent only on the object's height. Conversely, SLA and LFS printers utilize a laser to trace each point of the object individually. As a result, SLA and LFS printers generally match or exceed the speed of DLP printers when producing a single object or smaller items. However, DLP printers excel in speed when printing multiple objects that nearly fill the platform. Regarding the materials used in AM (also referred to as 3D printing), the choice varies depending on the printer model [9,12,13,17,43]. Some basic 3D printers are limited to producing diagnostic casts, while more advanced systems are capable of producing high-precision crown and bridge models, surgical guides, castable and pressable restorations, and biocompatible dental applications suitable for long-term use in the mouth, such as splints, orthodontic retainers, and dentures. While SLA, DLP, and LCD technologies use liquid photopolymers such as acrylates and epoxides, the 3D material extrusion technology (FFF) utilizes materials such as PLA or ABS. In contrast, PPP technology employs liquid photopolymer resins, primarily acrylates. Some 3D printers use only proprietary materials, restricting you to the resins supplied by the printer manufacturer. Other printers use an open system, allowing for the use of materials from different manufacturers. In 2022, Pereira et al. [45], in their study, utilized two distinct DLP printers to generate three distinct model sets, aiming to assess the impact of material choice on printing outcomes. While two groups adhered to the manufacturer's recommended resin, the third group employed a resin calibrated with a hand-held digital caliper. Findings indicated that deviating from the manufacturer's suggested resin significantly influenced the accuracy of the printed models.

The table below attempts a comparative evaluation of all systems based on this study (Table 1).

**Table 1:** Comparison of additive manufacturing technologies used for dental prosthetic models.

Technology	Printing Principle	Accuracy & Resolution	Surface Quality	Mechanical Properties	Cost & Accessibility	Typical Dental Applications
SLA (Stereolithography)	Laser-cured photopolymer resin	High (≈ ±50 μm)	Excellent, smooth	Moderate–High (material dependent)	Moderate–High	Prosthodontic models, implant planning, surgical guides
DLP (Digital Light Processing)	Projected UV light cures entire layer	Very High (≈ ±30–50 μm)	Excellent	Moderate–High	Moderate	Crowns & bridge models, orthodontic models, aligners
LCD (Masked SLA)	UV LED through LCD mask	High (≈ ±50 μm)	Very good	Moderate	Low–Moderate	Diagnostic & orthodontic models
FDM/FFF	Extruded thermoplastic filament	Moderate (≈ ±100–200 μm)	Fair–Poor	Low–Moderate	Low (high accessibility)	Study models, education, diagnostics
PolyJet (PPP)	Inkjet photopolymer droplets + UV curing	Very High (≈ ±20–30 μm)	Excellent	High (multi-material capable)	High	High-precision prosthodontic models, research

Limitations

Despite the growing body of evidence supporting the use of AM technologies in dentistry, several limitations should be acknowledged when interpreting the findings of the present narrative review. First, the majority of the included studies were conducted under in vitro or laboratory-controlled conditions. Although such settings allow for standardized evaluation of accuracy and trueness, they may not fully replicate the variability encountered in routine clinical practice, including differences in intraoral scanning quality, operator experience, and environmental factors.

Second, substantial heterogeneity exists among the reviewed studies with regard to printer types, materials, printing parameters, post-processing protocols, and evaluation methodologies. This variability limits direct comparisons across studies and complicates the establishment of definitive conclusions regarding the superiority of one AM technology over another.

Third, as a narrative review, the present study did not follow a systematic review protocol, and, therefore, the possibility of selection bias cannot be entirely excluded. Nevertheless, the aim of this work was to provide a comprehensive and clinically oriented overview of contemporary AM technologies rather than a quantitative synthesis of outcomes.

In addition, several inherent limitations should be acknowledged. Photopolymer-based materials used in SLA, DLP, and LCD technologies may exhibit long-term dimensional instability due to continued polymerization, moisture absorption, and thermal aging, which could negatively affect accuracy during prolonged storage or delayed clinical use. Furthermore, although many dental resins are certified as biocompatible, their suitability for long-term intraoral exposure remains limited compared to conventionally processed materials.

FDM-/FFF-printed models, while cost-effective and easily accessible, demonstrate inferior surface resolution and reduced mechanical strength compared to photopolymer-based systems, limiting their suitability for high-precision or high-stress prosthodontic applications. Additionally, variability in printer hardware, materials, slicing software, and post-processing protocols across studies restricts direct comparison of results and limits the generalizability of published findings.

Future directions

Future research in AM for dental applications should focus on addressing the current methodological and clinical gaps identified in the literature. High-quality prospective clinical studies are required to validate the accuracy and reliability of 3D-printed dental models under real-world conditions, particularly for complex prosthodontic and implant-supported restorations. Such studies should incorporate standardized workflows from intraoral scanning to final model fabrication.

The development and adoption of standardized protocols for accuracy assessment, including uniform reference models, measurement techniques, and reporting criteria, would significantly enhance comparability across studies and facilitate evidence-based decision-making. Additionally, further investigation into the long-term dimensional stability and aging behavior of printed models, especially those fabricated using photopolymer resins, is warranted.

Future research in dental AM should explore emerging technologies beyond conventional three-dimensional fabrication. Artificial intelligence-driven algorithms may optimize print orientation, layer thickness, exposure parameters, and support design, thereby improving dimensional accuracy and reducing material waste. In addition, the development of four-dimensional (4D) printing, in which printed structures respond dynamically to environmental stimuli, may enable adaptive orthodontic appliances and smart prosthetic components. Finally, increasing emphasis should be placed on sustainable and environmentally friendly materials, including recyclable polymers and energy-efficient printing workflows.

Ultimately, continued interdisciplinary collaboration between clinicians, dental technicians, material scientists, and engineers will be essential to fully realize the potential of AM technologies in modern dentistry.

## Conclusions

In our days, 3D printers are extensively utilized in both dental laboratories and clinics, capable of producing a variety of dental indications, including casts, surgical guides, splints, retainers, dentures, wax-ups, castable patterns, temporary and permanent restorations, and many other items. The findings of this paper indicate that SLA, DLP, and PolyJet technologies are among the most accurate options for creating full-arch dental casts for prosthodontic purposes, demonstrating high levels of precision.

The 3D production of dental models and related structures is an evolving process, with new developments emerging daily that improve the quality of the final products, as well as the time and cost of their production. Additional research is required to gain a deeper understanding of the accuracy and benefits of AM technology in dentistry.
